# The role of facial pattern variation for species recognition in red-fronted lemurs (*Eulemur rufifrons*)

**DOI:** 10.1186/s12862-018-1126-0

**Published:** 2018-02-13

**Authors:** Hanitriniaina Rakotonirina, Peter M. Kappeler, Claudia Fichtel

**Affiliations:** 1 Behavioral Ecology & Sociobiology Unit, German Primate Center, Göttingen, Germany; 2 Department of Sociobiology/Anthropology, Johann-Friedrich-Blumenbach Institute for Zoology, Georg-August University, Göttingen, Germany; 3 Wissenschaftskolleg zu Berlin, Wallotstr. 19, 14193 Berlin, Germany

**Keywords:** Red-fronted lemurs, Species recognition, Visual signals, Sexual selection, Genetic drift

## Abstract

**Background:**

Species recognition, i.e., the ability to distinguish conspecifics from heterospecifics, plays an essential role in reproduction. The role of facial cues for species recognition has been investigated in several non-human primate species except for lemurs. We therefore investigated the role of facial cues for species recognition in wild red-fronted lemurs (*Eulemur rufifrons*) at Kirindy Forest. We presented adult red-fronted lemurs pictures of male faces from five species including red-fronted lemurs, three closely related species, white-fronted lemurs (*E. albifrons*), brown lemurs (*E. fulvus*), rufous brown lemurs (*E. rufus*), and genetically more distant red-bellied lemurs (*E. rubriventer*), occurring in allopatry with the study population. We predicted that red-fronted lemurs respond stronger to conspecific than to heterospecific pictures and that females show stronger responses than males. In addition, if genetic drift has played a role in the evolution of facial color patterns in the members of this genus, we predicted that responses of red-fronted lemurs correlate negatively with the genetic distance to the different species stimuli.

**Results:**

Red-fronted lemurs looked significantly longer at pictures of their own species than at those of heterospecifics. Females spent less time looking at pictures of white-fronted, brown and red-bellied lemurs than males did, but not to pictures of red-bellied lemurs and a control stimulus. Individuals also exhibited sniffing behavior while looking at visual stimuli, and the time spent sniffing was significantly longer for pictures of conspecifics compared to those of heterospecifics. Moreover, the time spent looking and sniffing towards the pictures correlated negatively with the genetic distance between their own species and the species presented as stimulus.

**Conclusions:**

We conclude that red-fronted lemurs have the ability for species recognition using visual facial cues, which may allow them to avoid costly interbreeding. If so, sexual selection might have influenced the evolution of facial patterns in eulemurs. Since responses also correlated with genetic distance, our findings suggest a potential role of genetic drift as well as sexual selection in influencing the evolution of facial variation in eulemurs. Because study subjects looked and sniffed towards the presented pictures, red-fronted lemurs might have the ability for multi-modal species recognition.

**Electronic supplementary material:**

The online version of this article (10.1186/s12862-018-1126-0) contains supplementary material, which is available to authorized users.

## Background

The ability to differentiate conspecifics from heterospecifics plays an important role in reproduction [1–6]. Since females usually experience higher costs during reproduction than males, heterospecific mating is more costly for females [7–9]. Females should therefore be selected to recognize and discriminate against heterospecific males to avoid costly interbreeding [9]. Indeed, the ability for species recognition has been demonstrated in several taxa, such as bats using olfactory signals [6], fish using olfactory or visual signals [10, 11] and frogs, birds and mammals using acoustic signals [12–15].

Visual cues have been suggested to be important for several animal taxa as they can be used for individual as well as species recognition [11, 16–18]. In addition, facial color patterns are among the phenotypic traits that play a communicative role in many social interactions of primates [19–24]. Facial cues can contain visual information such as shape and colors that differ across individuals or species [25–28], and they can provide information about social status, condition and identity of an individual [22, 23, 29, 30]. Several studies have demonstrated the ability of non-human primates to differentiate individuals of their own kin/group from strangers and also to discriminate between conspecifics and heterospecifics based on visual cues [2, 21, 31–35]. For example, chimpanzees (*Pan troglodytes*) and rhesus macaques (*Macaca mulatta*) used facial cues in black-and-white photographs presented on a computer screen to discriminate between different individuals [22]. Chimpanzees are also able to discriminate kin by means of black-and-white photographs of mothers and their offspring, matching mother-son dyads but not mother-daughter ones [36]. Finally, Tonkean macaques (*Macaca tonkeana*) and brown capuchin monkeys (*Cebus apella*) were able to discriminate between pictures of conspecific and heterospecific individuals, as inferred by their longer looking time towards pictures of conspecifics [25].

The lemurs of Madagascar also exhibit highly diverse facial color patterns [37, 38], which may have a communicative function in social interactions as well as in species recognition. So far only a few studies have investigated the potential use of visual signals for individual or species recognition in lemurs, however. For example, brown and black lemurs (*Eulemur macaco*) were able to differentiate between familiar and unfamiliar individuals by using facial cues [39], and females of seven eulemur species differentiated colorful from non-colorful conspecific male photographs [40], suggesting a potential ability for visual species recognition as well. In contrast to visual signals, olfactory signals are used by some species to discriminate conspecifics from heterospecifics [41–43], whereas acoustic signals, especially long-distance calls, appear to be used in mouse lemurs (*Microcebus murinus*) to distinguish conspecifics from heterospecifics [44].

Among lemurs, eulemurs are the only taxon with sexual dichromatism, as males are particularly colorful and show considerably more variation in facial patterns than females [24, 38]. Eulemurs have dichromatic color vision, except for some females that exhibit polymorphic trichromacy [45–49], suggesting that variation in facial coloration can be perceived by them (see also [40]). Additional visual information used in this context may include variation in patterns, shape and contrasts.

**Fig. 1:**
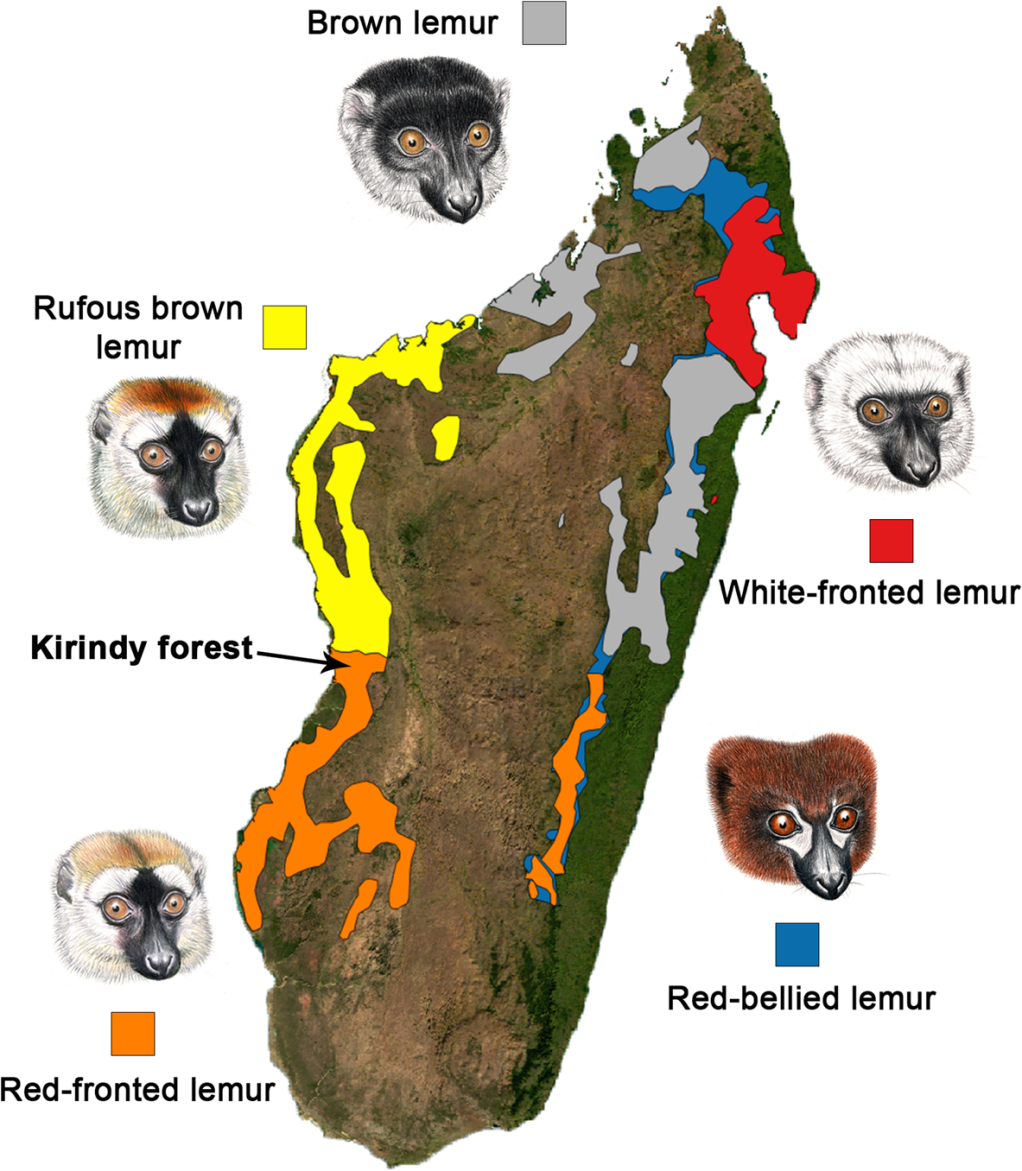
Map of Madagascar showing the distribution of *Eulemur* species used as stimuli during the experiments. The pictures depict drawings of the male faces of the different species used as stimuli. *Eulemur* illustrations provided by S. Nash

The ability to discriminate con- from heterospecifics based on visual cues is particularly important for species that live sympatrically with closely related species. Lemur communities can consist of up to 13 different species (e.g. in Andasibe, Ranomafana, Tsingy de Bemaraha [37]). Within the genus *Eulemur*, two congenerics occur in sympatry at several sites in Madagascar [37]. Additionally, eulemurs are known to form viable and sometimes fertile hybrids in captivity, and hybrids have been reported from a few natural contact zones [50–55]. Thus, it is biologically relevant to investigate whether lemurs have the visual capability to distinguish con- from heterospecifics, which can serve as one reproductive isolation mechanism to avoid costly interbreeding in the wild. Given the limited information available about the use of visual cues for species recognition in lemurs, despite their high diversity in pelage coloration and especially facial patterns, our study aimed to investigate the role of facial variation for species recognition in this radiation of primates. Specifically, we examined whether wild red-fronted lemurs (*Eulemur rufifrons*) can discriminate between different eulemur species that differ in their facial color pattern. We studied red-fronted lemurs in Kirindy forest, Western Madagascar [56, 57], where they do not occur in sympatry with any other eulemur species (see Fig. 1) [58]. We presented red-fronted lemurs a color photo of either a conspecific or heterospecific male, i.e., photographs of the closely related white-fronted, brown and rufous brown lemurs, which occur in allopatry, and the more distantly related red-bellied lemurs, which also occur in allopatry with the study population, but in sympatry with the eastern population of red-fronted lemurs (Table 1).

**Table 1:** Genetic distance and description of facial color patterns for the different species (species are listed according to their genetic distance between red-fronted lemurs and the respective species)

Red-fronted lemurs	Dark red crown, black muzzle, golden-red cheek beard, creamy-white patches above the eyes.
Rufous brown lemurs	Genetic distance: 0.35 Allopatric heterospecific and very similar to red-fronted lemurs in facial color patterns: brick-red crown, golden-red cheek beard, black muzzle and black midfacial stripe extending from crown to nose.
White-fronted lemurs	Genetic distance: 0.72 Occurs in allopatry with red-fronted lemurs and facial color variation differs strongly from red-fronted lemurs. Black muzzle and white beard, cheeks and crown.
Brown lemurs	Genetic distance: 0.72 Occurs in allopatry with red-fronted lemurs and is slightly different in facial color patterns. Dark-brown to almost black muzzle and crown, light grey beard and variable patches of light fur above the eyes.
Red-bellied lemurs	Genetic distance: 4.57 Occurs in sympatry with red-fronted lemurs in the eastern parts of Madagascar but not at the study site in the West and is very different in facial color patterns. Black muzzle, face shading to black; patches of white skin form characteristic “tear-drops” beneath the eyes, no bushy beard.

If variation in facial color patterns is used for species recognition in eulemurs, we predicted that red-fronted lemurs should respond stronger to pictures of faces of their own species than to pictures of faces of heterospecifics. Additionally, if sexual selection has played a role in the evolution of facial color pattern variation, we predicted that females should show stronger responses than males. Finally, as the species used as stimuli differ in phylogenetic distance to the test species, we predicted that if genetic drift has played a role in the evolution of facial color patterns in eulemurs, the response of red-fronted lemurs should correlate negatively with their respective genetic distance to the different species used as stimuli.

## Results

### Time spent looking towards the picture

**Fig. 2:**
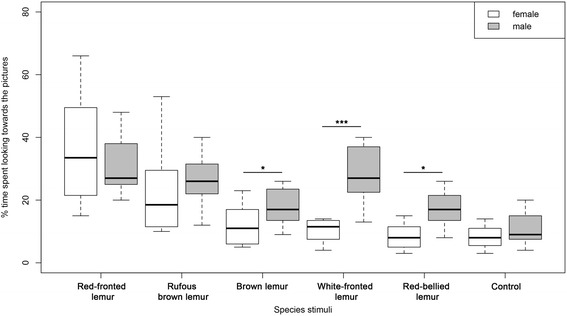
Boxplot of the percentage of time red-fronted lemurs spent looking towards the different stimuli showing the responses separated by sex. Depicted are the median (black bars), interquartile range (boxes) and ranges (whiskers)

Red-fronted lemurs looked significantly longer towards pictures of their own species than towards pictures of heterospecifics (Table 2, Fig. 2, LMM, X^2^ = 15.94, *p* < 0.01). Females spent significantly less time looking at pictures of white-fronted, brown and red-bellied lemurs than males did, but not at pictures of rufous brown lemurs, which are very similar in facial patterns, and the control (Fig. 2). Additionally, the percentage of time spent looking towards the pictures was significantly correlated with the genetic distance between red-fronted lemurs and the species providing the stimuli, but did not differ between the sexes. Red-fronted lemurs looked significantly longer at pictures of genetically more closely related congeners (Table 2, LMM, X^2^ = 21.69, *p* < 0.001).

**Table 2:** Parameter estimated for the Linear Mixed Models (LMM) on the influence of (a) the species of the presented picture and (b) the genetic distance between species on the percentage of time spent looking towards the pictures. The influence (c) of species of the presented picture and (d) the genetic distance between species on the percentage of time spent sniffing the pictures

	Model	Response variable	Random factors	Fixed factors	Estimate	SE	*P*-value
a	LMM	Percentage of time spent looking towards the pictures	Individual identity	intercept	0.64	0.04	< 0.001
				rufous brown lemurs	−0.16	0.05	< 0.01
				brown lemurs	−0.30	0.05	< 0.001
				white-fronted lemurs	−0.30	0.05	< 0.001
				red-bellied lemurs	−0.35	0.05	< 0.001
				control	−0.35	0.05	< 0.001
				sex	−0.05	0.06	0.47
				rufous brown lemurs-sex male	0.10	0.07	0.18
				brown lemurs- sex male	0.15	0.07	0.03
				white-fronted lemurs-sex male	0.26	0.07	< 0.001
				red-bellied lemurs-sex male	0.18	0.07	0.01
				control-sex male	0.09	0.07	0.21
b	LMM	Percentage of time spent looking towards the pictures	Individual identity	intercept	0.47	0.03	< 0.001
				genetic distance	−0.04	0.009	< 0.001
				sex	0.09	0.04	0.06
c	LMM	Percentage of time spent of sniffing events	Individual identity	intercept	0.34	0.04	< 0.001
				rufous brown lemurs	−0.10	0.05	< 0.05
				brown lemurs	−0.22	0.05	< 0.001
				white-fronted lemurs	−0.13	0.05	< 0.05
				red-bellied lemurs	−0.23	0.05	< 0.001
				control	−0.25	0.05	< 0.001
				sex	0.06	0.04	0.19
d	LMM	Percentage of time of sniffing events	Individual identity	intercept	0.25	0.04	< 0.001
				genetic distance	−0.03	0.01	< 0.01
				sex	0.06	0.05	0.27

### Duration of sniffing

**Fig. 3:**
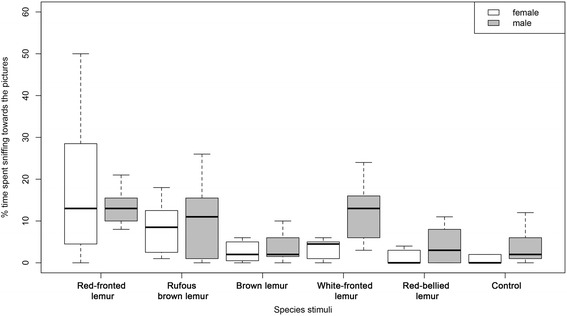
Boxplot of the percentage of time red-fronted lemurs spent sniffing the pictures. Depicted are the median (black bars), interquartile range (boxes) and ranges (whiskers)

Red-fronted lemurs also spent significantly more time sniffing towards pictures of their own species compared to those of all heterospecific stimuli. Sex did not influence the time spent sniffing towards the pictures (Table 2, Fig. 3, LMM, X^2^ = 32.92, *p* < 0.001). The percentage of time sniffing was also significantly correlated with genetic distance, with red-fronted lemurs sniffing significantly longer during presentation of photos of closely related congeners (Table 2, Fig. 3, LMM, X^2^ = 11.41, *p* < 0.01).

## Discussion

This study provides the first investigation of wild lemurs’ ability to discriminate between photographs of their own and closely related species. Our results indicate that red-fronted lemurs can use facial cues to discriminate between conspecifics and heterospecifics. Interestingly, test subjects also spent more time sniffing during presentation of conspecific pictures, indicating that they also may use olfactory cues in this context. Hence, they might simultaneously process olfactory and visual information to differentiate conspecifics from heterospecifics, suggesting that multi-modal communication might play a role in species recognition in these animals. Moreover, males and females differed in time spent looking towards the pictures of some heterospecifics, which may suggest a potential role of sexual selection in the evolution of facial variation in this species. Since the time spent looking as well as sniffing at the pictures were negatively correlated with genetic distances between red-fronted lemurs and the stimuli species, genetic drift may have also influenced the evolution of facial color patterns in this species. Finally, our study showed that the experimental use of photographs is feasible to test the ability of wild non-human primates for species recognition, which has been so far studied only in captive settings [2, 22, 34, 36, 39]. We discuss these key results in more detail below.

The variation in time spent looking at the pictures indicates that red-fronted lemurs are able to discriminate between the pictures of conspecifics and heterospecifics. Looking duration also appeared to correspond to the similarity of facial patterns between red-fronted lemurs and the other species: rufous brown lemurs are very similar in facial appearance to red-fronted lemurs, and these two species are also difficult for humans to distinguish (Fig. 1, Table 1), whereas white-fronted, brown and red-bellied lemurs are gradually more different in facial appearance. In addition, females spent less time looking towards pictures of white-fronted, brown and red-bellied lemurs than males, thus exhibiting a more pronounced differentiated response. Since we adjusted the size of the pictures to the average head size of the respective species, we cannot completely rule out the possibility that the responses of red-fronted lemurs were influenced by the slight differences in size of the pictures. However, if the size of the stimulus per se has influenced their responses, they should have responded more strongly to the control, which was adjusted to the average size of all stimuli, but they looked only briefly at this stimulus.

As interbreeding can occur in non-human primates (e.g. in macaques [59], eulemurs [50–55], sexual selection may act on species to avoid potentially costly heterospecific mating. Pronounced sexual dichromatism and striking differences in male patterning and coloration may provide a substrate for species recognition in the context of mate choice, and our experiments indicate that females perceive and respond to this variation. For example, red-bellied lemurs occur in sympatry with red-fronted, brown and white-fronted lemurs in the east of Madagascar [58]. Because these three species are visually very different from its sympatric congener, facial color variation might have played a role in creating reproductive barriers among these species during their recent divergence [60]. Because some eulemur species hybridize in their natural contact zones, future experiments in hybrid zones could investigate whether individuals in these zones discriminate potential mates based on variation in facial patterns.

Sex differences in responses towards the pictures might also reflect differences in color vision between sexes. Females can have polymorphic trichromacy or be dichromatic, whereas males are all dichromatic. Hence, eulemur females exhibiting a polymorphic trichromacy have the ability to perceive red and orange colors [61], and may therefore have shown a more pronounced differentiated response than males. However, the genetic tests required to test this assumption have not been performed. Interestingly, males payed more attention to stimuli of white-fronted, brown and red-bellied lemurs than females. Indeed, facial colors of these three species are dominated by a dark face with light (white or light gray) patches (Fig. 1). As red-fronted lemur males exhibit only dichromatic color vision [24, 62], contrasting dark and light areas might be more salient stimuli to them than to potentially trichromatic females.

The degree of phenotypic differences between red-fronted lemurs and white-fronted, brown, rufous brown and red-bellied lemurs also corresponds both to the genetic distance between them, as well as to the time red-fronted lemurs spent looking at the various stimuli. Similarly, the looking duration of macaques towards pictures of several heterospecific species also correlated with their morphological similarity in facial patterns as well as the genetic distance between them [2, 34]. Genetic differentiation as a result of drift during and following recent speciation events may therefore also have played a role in the evolution of facial color pattern in eulemurs.

Finally, multiple studies demonstrated that animals can process and use signals of different modalities for species recognition [63–65]. For example, male blackcaps (*Sylvia atricapilla*) are able to associate acoustic and visual sensory modalities in matching species-specific songs and species-specific plumage to distinguish their own species from sympatric heterospecifics (*Sylvia borin*) during playback experiments presented in combination with stuffed models of conspecifics and heterospecifics [64]. In non-human primates, for instance, tufted capuchin monkeys (*Cebus apella*), rhesus macaques (*Macaca mulatta*) as well as Japanese macaques (*Macaca fuscata*) are able to use visual and acoustic sensory modalities (voice-face matching) to distinguish between conspecifics and heterospecifics [66–68]. Moreover, ringtailed lemurs (*Lemur catta*) are capable of multi-modal (olfactory-auditory matching) individual recognition [69], and the use of olfactory signals for species recognition in some eulemurs has been already shown [41, 43]. Whether red-fronted lemurs are capable of multi-modal species recognition was not explicitly tested in this study. However, our results showed that while red-fronted lemurs processed visual cues during the experiment, they also sniffed at the stimuli. Thus, red-fronted lemurs might be able to use two different sensory modalities (olfactory-visual matching) at the same time to discriminate individuals of their own species from heterospecifics. However, explicit experiments with signals of two different modalities are required to confirm our preliminary conclusion that red-fronted lemurs dispose of multi-modal species recognition abilities.

## Conclusions

This study revealed the importance of facial cues as visual signals for species recognition in wild red-fronted lemurs. Females of red-fronted lemurs may also be better at differentiating conspecifics from heterospecifics due to sex difference in color vision abilities. Our findings suggest a potential role for sexual selection as well as genetic drift in influencing the evolution of facial variation in eulemurs. Moreover, this study revealed evidence for visual species recognition abilities in wild red-fronted lemurs and also suggested a potential for multi-modal species recognition. However, it remains unclear which specific components of the facial cues are used for species recognition, requiring further investigations to identify the essential cue(s), such as colors, patterns or a combination of both, used by eulemurs to discriminate their own from different species as well as among individuals.

## Methods

### Study site

Experiments were conducted with red-fronted lemurs in Kirindy Forest, western Madagascar (Fig. 1). Study subjects are individually marked as part of a long-term study and are well habituated to human observers [56, 57]. We studied eight adult females and seven adult males in four different groups (2–5 subjects per group).

### Experimental design

**Fig. 4:**
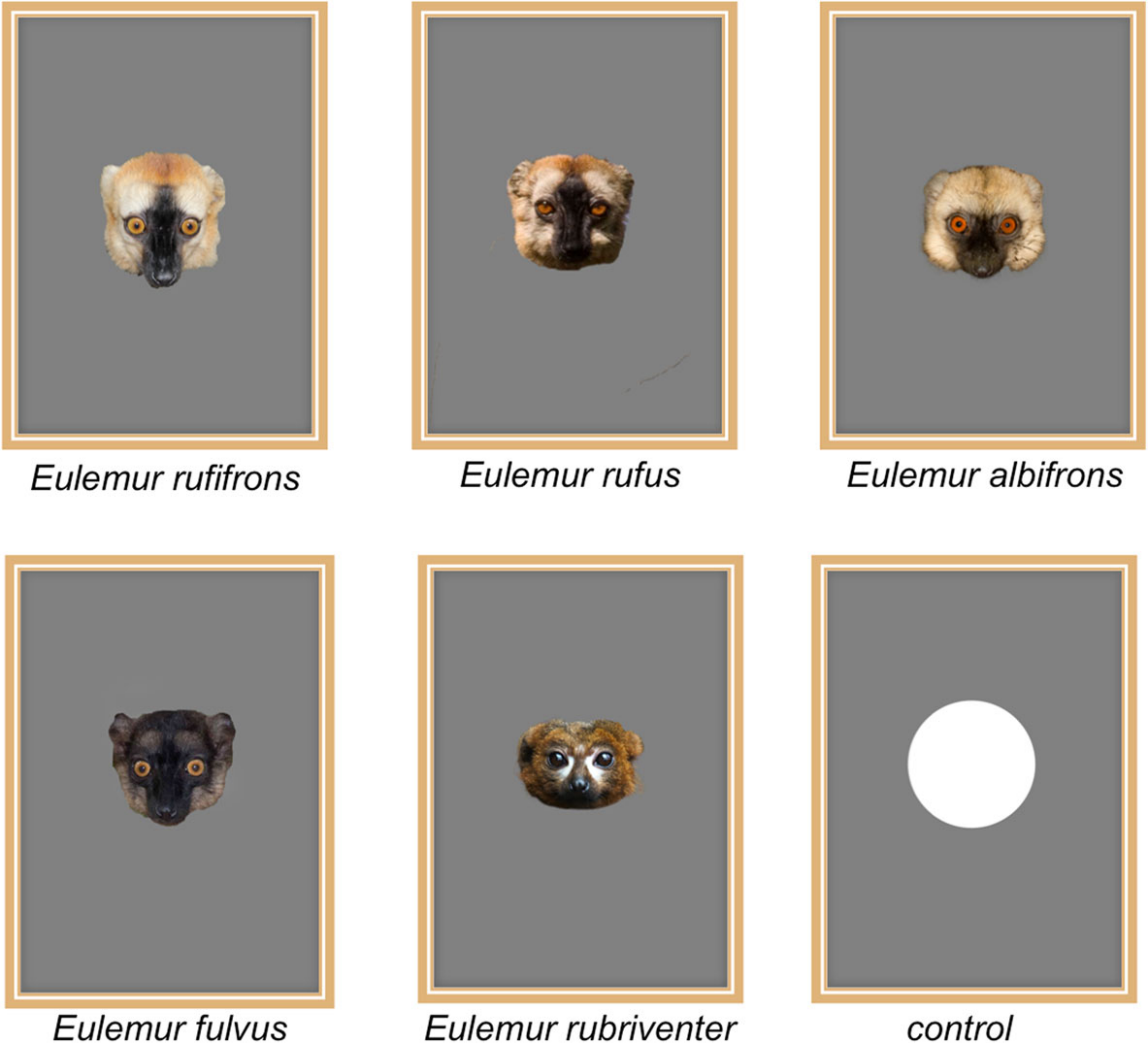
Examples of pictures of each species used as stimuli during the experiments and the control (white circle). *Eulemur* photographs: M. Markolf

During the experiments, we presented each red-fronted lemur a color photo of either a conspecific or heterospecific male, i.e., photographs of a red-fronted lemur, the closely related red, brown and white-fronted lemurs and of the more distantly related red-bellied lemurs. Species used as stimuli were chosen according to information on genetic distance, visual appearance and geographic distribution (see Table 1 and Fig. 1). Each photograph contained only the head of the animal on a gray background (Fig. 4) and was adjusted to have approximately the same size (head length and width) as the head of the given species. As a control, we presented a picture frame containing a white circle on a gray background having the average size of the faces on the other pictures (Fig. 4). Each picture was placed in a picture frame made of wood to facilitate the presentation of the picture to the focal animal as well as to stabilize the picture itself (Fig. 4). Variation in facial color patterns of the species used as stimuli during the experiments is provided in Table 1, based on descriptions in Mittermeier et al. [37].

**Fig. 5:**
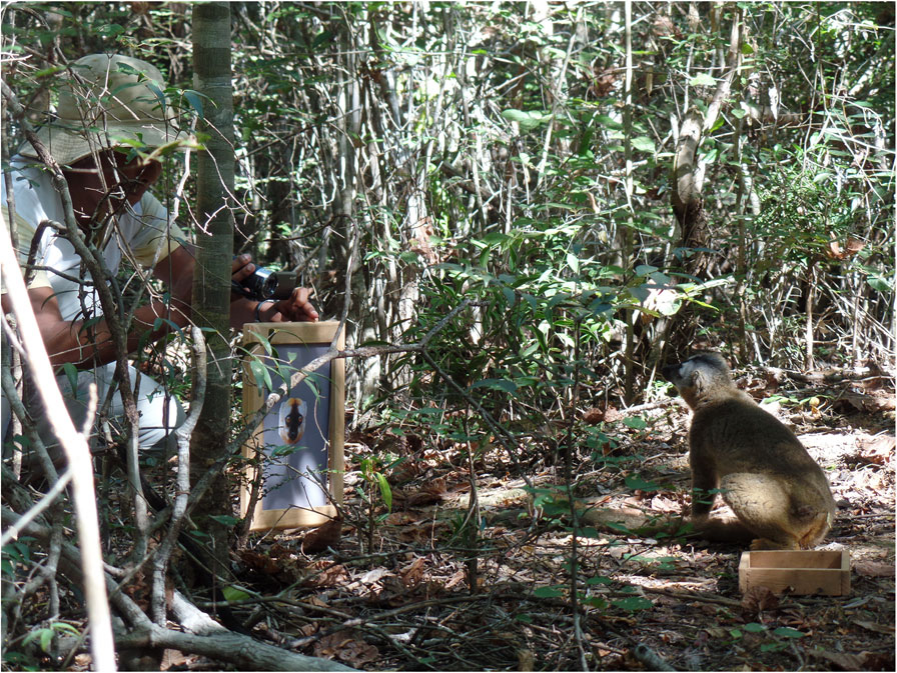
Photograph showing the procedure of an experiment

Before each experiment, individuals were attracted with an acoustic signal to a location on the ground, where they were fed some raisins (see for detailed protocol of the training Schnoell & Fichtel [70]). The experiment was started when the focal subject finished feeding, and was engaged in quiet activities such as resting or grooming at the periphery of the group. The experimenter (HR) approached the focal individual carefully by hiding the picture frame behind the back until the focal individual was stationary on the ground. Then the picture was presented at a distance of about 1 m in front of focal individual at the same height as the focal individual (Fig. 5). We presented only one picture and not two pictures simultaneously, because with such a paired design, we would have had to present the pictures of their own species repeatedly, which might have caused habituation. In order to avoid pseudo-replication, every individual was tested with a picture of a different, unknown individual of the given species, and pictures were presented in a randomized order. Each individual was tested only once every second day. All experiments were conducted during the breeding season of red-fronted lemurs.

Responses of experimental subjects were recorded with a SONY digital video camera from briefly before until 60 s after the onset of each experiment. The camera was placed in front of the focal animal, aligned with the picture frame so that looking direction could be clearly recorded. Based on these video-recordings, we measured the time each subject spent looking towards the picture after the onset (looking direction within a 45° angle of the direct line of sight towards the picture), and calculated the percentage of time spent looking towards the picture from the total time spent looking around. In addition, during the experiments, we observed sniffing behaviors of focal individuals while conducting the experiments. We therefore measured also the time individuals spent sniffing (inhaling a short and distinct breath through the nose combined with very small movements of the snout) towards each picture after the onset and calculated the percentage of time spent sniffing (see Additional file 1). Videos were analyzed frame-by-frame with a resolution of 30 frames/s, using Adobe Premiere Elements (12.0). All experiments were rated by HR, and 10% were rated again by a second observer naïve to the research question. We used R to calculate the intra-class correlation coefficient (ICC) to test for inter-observer reliability. The resulting ICC was 0.95 indicating strong agreement between raters.


Additional file 1:Sample video showing an individual of red-fronted lemurs looking at the picture with sniffing behavior. (MP4 3840 Kb)


### Statistical analyses

We used linear mixed models (LMM) to test for differences in the percentage of time red-fronted lemurs spent looking towards the pictures as well as the percentage of time spent sniffing at the pictures in response to different stimuli using LmerTest package in R [71]. Percentage of time looking towards the pictures and percentage of time sniffing in the direction of the pictures were arcsine-square root transformed and fitted as responses. Species and sex were fitted as fixed factors and individual identity as a random factor to control for repeated measurements. Because the genetic distance correlates with the categories of the species, we fitted a second LMM in order to examine whether the percentage of time red-fronted lemurs spent looking and sniffing towards the pictures was influenced by the genetic distances between red-fronted lemurs and the species providing the stimuli. The percentage of time spent looking or sniffing towards the pictures were fitted as response, genetic distance and sex were fitted as fixed factors and individual identity as random factor. All analyses were conducted in R version 3.1.3.
